# Unconscious goal pursuit strengthens voluntary force during sustained maximal effort via enhanced motor system state

**DOI:** 10.1016/j.heliyon.2024.e39762

**Published:** 2024-10-30

**Authors:** Yudai Takarada, Daichi Nozaki

**Affiliations:** a Faculty of Sports Sciences, Waseda University, Saitama 359-1192, Japan; b Graduate School of Education, The University of Tokyo, Tokyo 113-0033, Japan

## Abstract

Maximal voluntary force is known to be enhanced by shouting during sustained maximal voluntary contraction (MVC) via the enhancement of motor cortical excitability. However, whether excitatory input to the primary motor cortex from areas other than the motor-related cortical area induces muscular force-enhancing effects on the exertion of sustained maximal force remains unclear. Therefore, by examining motor evoked potentials to transcranial magnetic stimulation during sustained MVC and assessing handgrip force, the present study aimed to investigate the effects of subliminal goal-priming with motivational rewards on the state of the motor system. The findings revealed that when combined with rewards in the form of a consciously visible positive stimulus, barely visible priming of an action concept increased the maximal voluntary force and reduced the silent period (i.e., reduced motor cortical inhibition). To our knowledge, this is the first study to report a link between the muscular force of subliminal reward-goal priming during MVC and the enhancement of motor system activity through subliminal reward-goal priming operating on the motor system, possibly through the potentiation of activity of the reward-linked dopaminergic system.

## 1 Introduction

In human performance, muscle fatigue can be defined as any exercise-induced decrease in maximal voluntary force exerted by a muscle or muscle group [1,2]. During muscle fatigue, motor system activity induced by maximum volition does not induce sufficient drive in the motoneurons to exert the full force of which the muscles are capable [3,4]. However, whether events in the primary motor cortex (M1) itself contribute directly to this failure (i.e., supraspinal fatigue), or whether supraspinal fatigue occurs because of decreased excitatory input from other areas to the M1, including subcortical regions such as the basal ganglia (BG) in the limbic structures, remains unclear. Thus, the behavior of neuronal cells in the M1 during maximal voluntary contraction (MVC) in lethargizing maximal effort remains unclear.

Recent studies have reported that a self-produced shout elevated maximal voluntary handgrip force during sustained lethargizing maximal effort [5] with a shortened silent period, irrespective of changes in background electromyography (EMG) signals, which have been used as an index for evaluating motor excitability [6]. These results suggest that the muscular force-potentiating effect of shouting during handgrip force production with maximal effort may be linked to the enhancement of motor system activity generated by the additional drive of shouting acting on the M1. Thus, this phenomenon may reflect not only an internal increase of activity in the M1, but also an effect of motor-related cortical excitatory input to the M1 on supraspinal fatigue. Neural activity in the limbic circuit that starts and ends in the anterior cingulate area and medial orbitofrontal cortex (OFC) and operates on the M1, i.e., the segmented basal ganglia–thalamocortical circuit, would be expected to diminish motor cortical suppression during sustained MVC in lethargizing maximal effort if the additional neural activity operating on the M1 during sustained lethargizing maximal effort diminished motor cortical suppression. The limbic system, BG, OFC, thalamus (TH), prefrontal and anterior cingulate cortices, premotor and supplementary motor areas, and M1 are interconnected via a re-entrant neural circuit, thereby constituting an acceleration system for enhancing the motor output from the M1 against supraspinal fatigue [[7], [8], [9], [10], [11]]. Motivational inputs to this facilitation system have been shown to enhance M1 activity, thereby elevating the motor output to the peripheral system [12]. Priming stimuli with rewards could be expected to increase maximal voluntary force through the enhancement of the motor system state during sustained fatiguing maximal effort exertion, such as in brief non-fatiguing maximal effort exertion [13]. Regarding the processing of reward stimuli, this is accomplished by limbic structures connected to frontal areas in the cortex such as the nucleus accumbens and ventral striatum, which promote goal pursuit and play a central role in determining the reward value of outcomes [14].

Given the anatomically and functionally close connections between the dopaminergic and noradrenergic systems [14], this affective-motivational effect on the motor system state could be associated with both the reward-related dopaminergic and the pupil-linked neuromodulatory systems [15]. The locus coeruleus (LC) is known to regulate the interaction between these systems and their mutual connections to the prefrontal cortex [14]. Pupil dilation has frequently been utilized as an indirect index of LC activity because the release of noradrenaline from the LC leads to an increase in pupillary size via the α2-receptors [[15], [16], [17]]. An association between pupillary dilation and cognitive load has been demonstrated in paired-associate learning [18,19] and imagery tasks consisting of both abstract and concrete words [[20], [21], [22]]. In addition, pupillary changes under constant luminance have long been used as an index of cognition-related central autonomic activities, such as attentional effort [[23], [24], [25]].

In the present study, we investigated the effects of unconscious goal pursuit on the motor system state by testing motor evoked potentials (MEPs) using transcranial magnetic stimulation (TMS) applied over the hand area of the contralateral M1 while presenting motivational rewards via the manipulation of barely visible goal-priming cues during sustained MVC in lethargizing maximal effort. We also investigated the pupil-linked neuromodulatory system state by testing pupil size [26,27], and motor action by estimating handgrip maximal voluntary force [13,27]. When TMS is applied over the M1 during voluntary contractions, EMG shows MEPs accompanied by a period of near silence lasting more than 200 ms with a high-intensity stimulus [28,29]. The initial part of this silent period (from about 50 to 80 ms) might represent both the suppression of the descending drive and diminished motoneuronal activity. Moreover, the latter and final parts of the silent period might reflect the intracortical inhibition reported to be observed in paired-pulse testing and epidural anesthesia both at rest and during voluntary contraction [30,31]. A previous study on the biceps brachii found that the silent period increased by more than 50 ms during a 2-min sustained MVC [[31], [32]]. Given that suppression within the cortex has been shown to lead to the latter part of the silent period following TMS over the M1 [28,33,34], this increased silent period indicates increased cortical suppression [35]. Given this background, in the present study, we measured the TMS-evoked duration of the silent period and adopted its corresponding absolute value as an index of the degree of intracortical suppression.

Our results indicated that barely visible goal-priming with motivational rewards reduced the silent period during the production of sustained maximal force and elevated handgrip force with maximal effort, which was accompanied by pupillary dilation. Thus, the muscular force-enhancing effect of unconscious goal pursuit in maximal force production appears to be related to the increase of motor system activity induced by the additional drive of unconscious goal pursuit acting on the M1. This new insight into the mechanisms underlying the unconscious goal pursuit-induced potentiation effects on force exertion may have considerable applications. For example, it may be possible to utilize the muscular force-potentiating effects of unconscious goal pursuit on maximal force exertion via exposure to positive words combined with the representation of a particular behavior with or without awareness when an individual is required to exert muscular force with maximal effort in an environment in which shouting is inappropriate (e.g., rehabilitation institutes, gymnastics). Specifically, coaches and physical therapists could consciously utilize the muscular force-potentiating impacts of unconscious goal pursuit on sustained fatiguing voluntary contraction including maximal force production to improve athletic performance and patients’ symptoms, irrespective of whether athletes and/or patients do this consciously.

## 2 Materials and methods

### 2.1 Power analysis

To determine the necessary sample size for the experiment, we conducted a priori power analysis. We designed the experiment to have 80 % power for detecting the effect sizes previously identified regarding the effects of motivational goal-priming on the motor system (0.64, Cohen's *d*) and motor action (0.50–0.82, Cohen's *d*) [13] and/or pupil diameter (0.61, Cohen's *d*) [27]. The significance level was set to 5 %. G∗Power 3.1® (Institut für Experimentelle Psychologie, Düsseldorf, Germany) was used to calculate the required total sample size with a repeated-measures analysis of variance (ANOVA) and within-subject factors (control or priming or priming + reward), using 95 % power (1–β error probability). The required sample size was 12 participants.

### 2.2 Participants and procedures

The study participants were 17 healthy Japanese right-handed university students (15 men, 2 women; mean age ± standard deviation [SD], 19.3 ± 0.95 years; mean height, 168.0 ± 4.9 cm; mean weight, 62.4 ± 11.1 kg; mean body mass index, 22.1 ± 3.8 kg/m^2^) who were evaluated using the Edinburgh Handedness Inventory [36]. No neurological, psychiatric, or other contraindications to TMS were reported [37]. All the participants had either normal or corrected-to-normal visual acuity in both eyes, and none were aware of the purpose of the study. Written and verbal informed consent was obtained from all participants. This study was carried out in accordance with the Declaration of Helsinki of 1964, as revised in 2013. Pregnant women were excluded to circumvent the unknown effects of TMS on fetuses. None of the participants reported having a history of resistance training, indicating that they had not received training in the production of the maximal force by a muscle or muscle group.

**Fig. 1:**
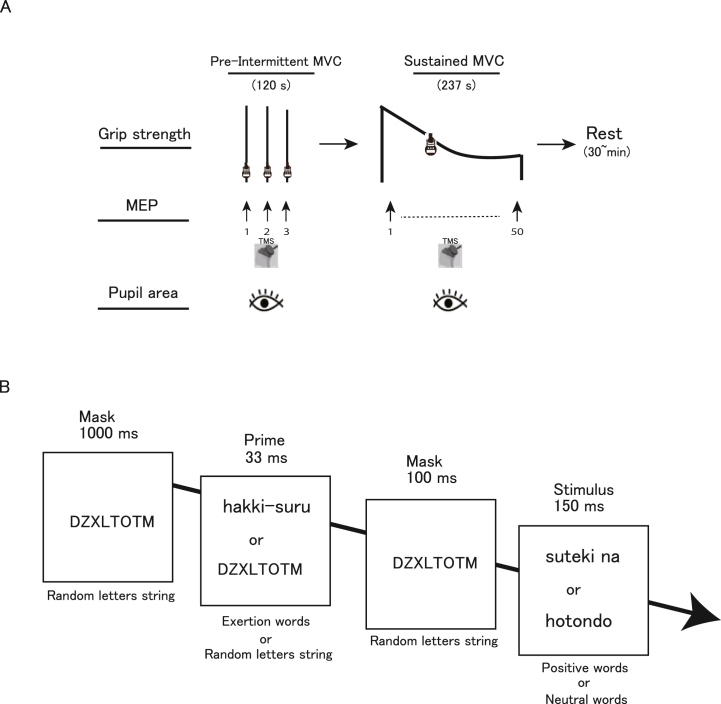
Experimental and priming procedures. (A) Experimental procedure. Each experiment comprised two tasks (pre-intermittent maximal voluntary contraction [MVC] and sustained MVC), which were spaced at least 3 min apart on the same day. The pre-intermittent MVC task was used as a baseline for the sustained MVC task, which consisted of three conditions (control, priming, and priming-plus-reward), each with a duration of approximately 237 s. Each condition was performed with an interval of more than 30 min. The total experimental time exceeded 90 min. The timing of transcranial magnetic stimulation (TMS) is indicated by the arrow. (B) Priming procedure. In the priming-plus-reward condition, the subliminal barely visible exertion primes were always paired with positive words. In the priming condition, although exertion primes and positive words were both displayed, they were never paired with each other. In the control condition, subliminal barely visible exertion words were never displayed. The order of possible stimulus pairs was randomized within each condition. Exertion, positive, and neutral words were shown in Japanese. Each trial in each condition began with a 1000-ms presentation of a random eight letter string (e.g., DZXLTOTM) as a forward mask. This was followed by the subliminal barely visible prime, displayed for 33 ms. A random letter string was again displayed for 100 ms as a backward mask, after which a consciously visible word was presented for 150 ms. Occasionally, a dot was presented for 33 ms (the dot was visible because of the absence of a backward mask), either above or below the neutral or positive word.

The experiments were devised with the aim of elucidating the effects of motivational goal-priming on sustained handgrip maximal voluntary force, pupillary size, and MEPs in the flexor carpi ulnaris (FCU) muscle in response to TMS. The reason we chose to examine the FCU muscle is that electrodes attached to this muscle are not strongly affected by the palms and/or palm motions, and are unlikely to contact the handgrip device; therefore, electrical signals can be obtained with less noise. The amplitude of MEPs in the FCU muscle has also been found to increase with increased intensity of brief isometric handgrip contractions (up to 80 % MVC) [38]. Additional motor units are recruited as the intensity of voluntary contractions increases up to 80 % MVC. In this study, each experiment comprised two tasks (pre-intermittent and sustained MVC) with intervals of at least 3 min on the same day. The experimental period was approximately 90 min in total. In the pre-intermittent MVC task, which served as a baseline for the sustained MVC task, the participants carried out three brief non-fatiguing MVCs (duration: 2–3 s) after being given a cue (“one, two, three, squeeze”) by the experimenter with a 60-s inter-squeeze interval. The stimulus intensity applied in the sustained MVC tasks could be ascertained and was sufficiently high for a single MEP waveform for discrimination against background EMG (bEMG) activity during voluntary activation in brief non-fatiguing maximal effort. After a rest period of approximately 3 min, the participants carried out sustained MVC in accordance with the experimental instructions, which were displayed on a monitor in front of them. No visual feedback regarding handgrip force or verbal encouragement was provided during three brief MVCs in the pre-intermittent MVC task or the sustained MVC in the sustained MVC task. The sustained MVC task was performed under three conditions, each lasting approximately 237 s, during the same session (Fig. 1A). No participants were told in advance about when the sustained MVC condition would end. The following three conditions were tested: a priming-plus-reward condition, in which priming words related to physical exertion were paired with “reward” words; a priming condition, in which priming words were paired with non-reward words; and a control condition, which contained no priming words. The participants were subjected to all three conditions in random order with an interval of more than 30 min between conditions to allow the recovery of central fatigue [39,40]. For this reason, as a baseline for the sustained MVC task, the participants were asked to perform three brief MVCs in the pre-intermittent MVC task. The numbers of participants in the six possible combinations of the execution order were as follows: control, priming, priming-plus-reward (n = 2); control, priming-plus-reward, priming (n = 2); priming, control, priming-plus-reward (n = 3); priming, priming-plus-reward, control (n = 3); priming-plus-reward, control, priming (n = 3); and priming-plus-reward, priming, control (n = 4). To evaluate motor cortex excitability, TMS was applied to the left M1 at 1.5 s after the disappearance of a positive or neutral word. Accordingly, 50 MEPs were obtained for each condition, with 153 total MEPs (TMS) for each participant in the two experimental tasks (pre-intermittent and sustained MVC). The participants were instructed to keep their heads still and their hands on their lap in a sitting posture while maintaining as much core stability as possible under all conditions, and to keep viewing the monitor in front of them.

### 2.3 Testing of subliminal stimuli

To confirm that the subliminal primed words were not consciously perceived, a separate experiment where different participants (36 males, 8 females; mean age ± SD: 22.0 ± 2.0 years) completed a task under both the subliminal and supraliminal conditions was conducted. In this experiment, the participants were instructed to indicate whether they saw a Japanese verb related to physical exertion. Post-masked subliminal exertion-related primes were not reported. Their response accuracy was 50.09 % ± 0.94 %, indicating that their judgments did not significantly differ from chance and that they were unable to see the priming words.

### 2.4 Priming procedure

The experimental procedure used in a previous study [41] to induce enhancement of the motor system state [26,27] was utilized to manipulate unconscious goal pursuit (Fig. 1B). Five Japanese verbs related to physical exertion (“to exert” [*hakki-suru*], “to struggle” [*funtou-suru*], “to work hard” [*mogaku*], “to energize” [*sei wo dasu*], and “to strive” [*doryokusuru*]) were used as motor goals, five positive adjectives (“nice” [*suteki na*], “great” [*subarashii*], “fantastic” [*kibunsaikouno*], “satisfactory” [*manzoku na*], and “enjoyable” [*tanoshii*]) were used as positive reward, and five neutral adverbs (“almost” [*hotondo*], “at least” [*sukunaku tomo*], “finally” [*saigoteki ni*], “nearly” [*hobo*], and “already” [*sude ni*]) were used as non-reward words. Under the priming-plus-reward condition, a barely visible presentation of one of the five exertion words was followed by the fully visible presentation of one of the five positive words in 25 of the 50 trials. In the remaining 25 trials, a barely visible presentation of a random letter string was followed by the fully visible presentation of one of the five neutral words. Therefore, in this condition, the barely visible exertion primes were always paired with positive words. In the priming condition, the exertion primes were paired with neutral words in 25 trials, while the random letter strings were paired with positive words in the other 25 trials. Therefore, in this condition, exertion primes and positive words were both displayed but never paired. In the control condition, only random letter strings were used as primes; these were paired with positive and neutral words in 25 trials each, and barely visible exertion words were never displayed. Consequently, positive and neutral words were balanced, with 25 trials each across all conditions. In addition, possible stimulus pairs were presented in random order under each condition. By designing the three different conditions this way and conducting comparisons among them, the affective shaping effects of associating behavioral states (exertions words) with positive affect (positive words) could be elucidated.

Each trial under each condition started with a presentation (duration: 1000 ms) of five different strings of eight pseudorandom letters (DZXLTOTM, YSTZBXTU, VCFTHYPC, CBEXGTVY, and ZTAWYDBH) as a forward mask (Fig. 1B), followed by the barely visible prime (duration: 33 ms). One randomly selected string from these five strings was then displayed again (duration: 100 ms) as a backward mask, followed by a consciously visible word (duration: 150 ms). A dot was also randomly presented for 33 ms (visible because of the absence of a backward mask) above or below the neutral or positive word. In accordance with a previous study [41], the participants were asked to indicate whether they had seen the dot, which served to bring the post-masked, barely visible primes to their attention. Trials were conducted every 3.5 s under each condition. The words were displayed on a 60-Hz cathode ray tube screen, and the experimental procedure was created using software designed for psychological experiments (Inquisit 3 Desktop Edition; Millisecond Software, Seattle, WA, USA).

### 2.5 Pupil diameter measurement and analysis

To measure the diameter of the participants’ pupils, we used an existing method for examining the effects of unconscious goal pursuit on the pupil-linked neuromodulatory system state [26,27]. The pupil diameter of the right eye was measured using the TalkEye Lite system (Takei Scientific Instruments, Tokyo, Japan). Images around the pupil were obtained using a camera equipped with near-infrared light-emitting diodes and a video graphics array (640 × 480) (built-in digital signal processor) camera module (NCM03-V; Nippon Chemi-Con Corporation, Tokyo, Japan). Banalization processing was performed on each image, after which, the pupil diameter was measured [42]. Changes in pupil size were estimated based on the pupil area [26,27] while the participants were viewing the monitor in front of them across all conditions. The average pupil area (dots) was calculated for 500 ms before each TMS while the participants squeezed a handgrip device across all three conditions of the sustained MVC task.

The following steps were taken to exclude the effects of experimenter expectations on the participants’ responses and measurements and estimate objectively the effects of motivational goal-priming under the sustained MVC task. First, the experimental procedure for the sustained MVC task was carried out automatically using a 60-Hz cathode ray tube screen to display the text, and the experimental procedure was designed using specialized software (Inquisit 3 Desktop Edition; Millisecond Software). Second, all participants were asked to follow the starting and stopping signals displayed on the monitor. Third, the measurements of pupil diameter were performed automatically using a specially designed device that included an eye-capturing camera. As a result, the paradigm used in this study was considered to be less susceptible to experimenter bias than the outcome measurements typically used to examine maximal voluntary force [43].

In the experimental procedure, all word stimuli were displayed in black (mean value of five measurements of luminance using a luminance meter: 22.5 cd/m^2^ [LS160; Konica Minolta, Tokyo, Japan]) on a white screen (124.3 cd/m^2^). Immediately before the presentation of the word, the screen color was momentarily white without any black words. Thus, the diameter of the pupil may have decreased as a result of the increase in luminance caused by the white screen (maximum luminance: 129.6 cd/m^2^). Consequently, the possibility that this transient change in luminance affected pupil diameter could not be completely eliminated. However, any effect on the results was considered to be minimal because this phenomenon was present for all participants across all conditions.

### 2.6 Handgrip force measurement and analysis

**Fig. 2:**
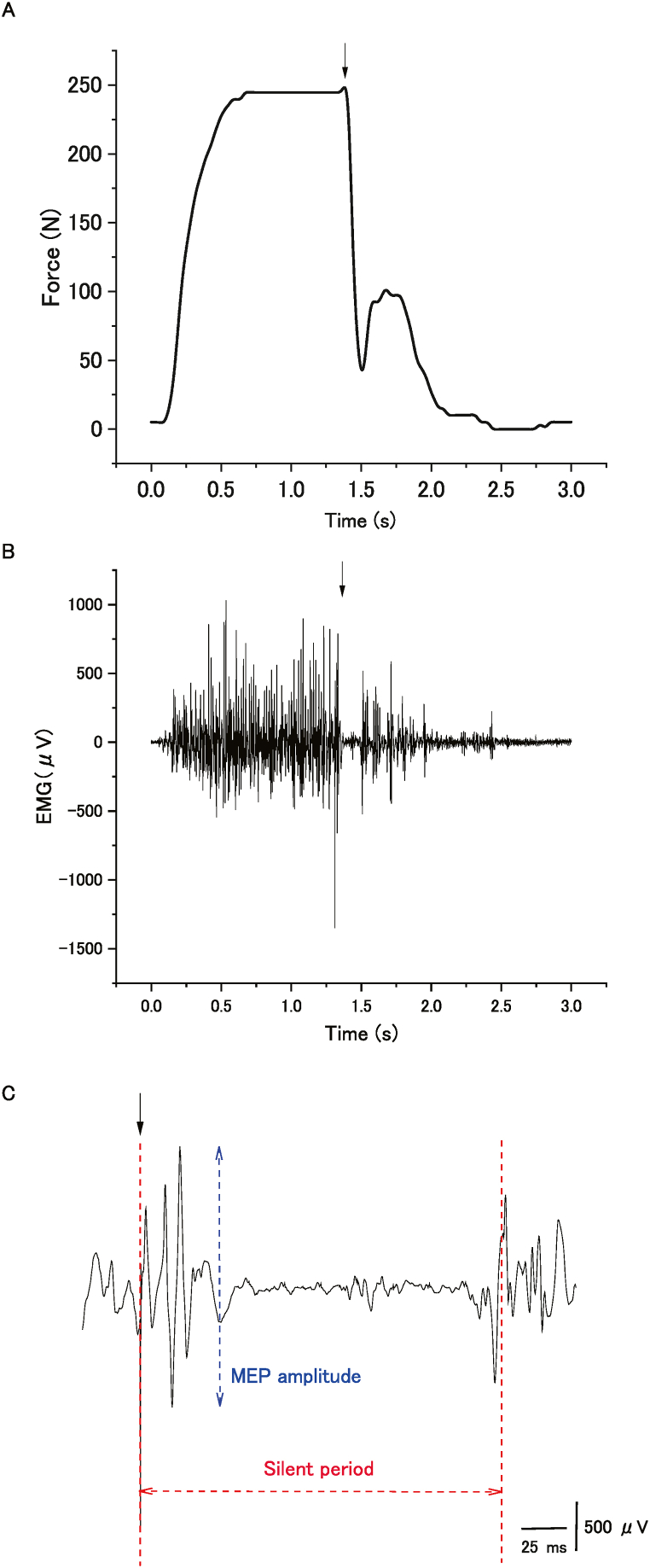
Typical recordings of handgrip force, and background electromyography (bEMG) and of motor evoked potential (MEP) waveforms of the flexor carpi ulnaris during the maximal voluntary contraction (MVC) of handgrip in a pre-intermittent MVC task in a single participant. The timing of transcranial magnetic stimulation (TMS) is indicated by the arrow. The handgrip force declined when TMS was delivered during the contraction, the timing of which was different in each contraction. (A) Handgrip force, (B) bEMG, and (C) MEPs during handgrip MVC. The bidirectional arrows indicate amplitudes of MEP (blue) and the silent period (red).

**Fig. 3:**
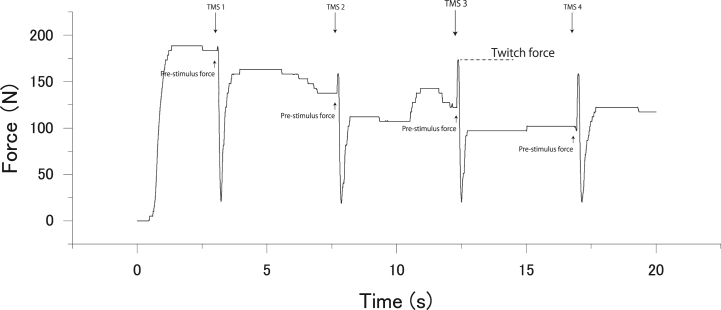
Typical recordings of handgrip force during the maximal voluntary contraction (MVC) of handgrip in sustained MVC task in a single participant. The timing of transcranial magnetic stimulation (TMS) is indicated by the arrow. Handgrip force produced by the superimposed twitch (twitch force) following TMS was expressed as a fraction of the pre-stimulus force at each TMS.

Voluntary force was measured using a handgrip device with a strain gauge (KFG-5-120-C1-16; Kyowa Electronic Instruments, Tokyo, Japan). This device was amplified (AD240-A; TEAC Instruments, Kawasaki, Japan), digitized (4 kHz), and filtered using a Butterworth filter (cutoff frequency: 10 Hz). Prior to the pre-intermittent MVC task, the participants practiced handgrip force exertion several times. The intensity of the applied stimulus was determined in the experimental tasks. Specifically, the participants were asked to maintain contraction for at least 1 s after the stimulus because continuing stable muscular contraction after the delivery of a TMS pulse is considered important to enable the accurate marking of the point of resumption of pre-stimulus EMG activity during active tonic muscular contraction. During the pre-intermittent MVC task, the participants performed three brief MVCs (duration: 2–3 s) after being given a cue (“one, two, three, squeeze”) by the experimenter, with 60-s inter-squeeze intervals. More specifically, the participants were asked to maintain contraction for at least 1 s after TMS. In the sustained MVC condition, the experimenter asked the participants to squeeze the handgrip device with the right (dominant) hand with maximum effort after seeing a priming word on the display and to stop squeezing when the word disappeared. An elastic band was used to fix the handgrip device to the right thigh so that it did not move when squeezed. The maximal exerted force values were averaged from the 500-ms steady state of the force curve before each TMS (Fig. 2A) across all three trials for the pre-MVC condition with three brief MVCs and across all 50 trials for the sustained MVC condition with 50 MEPs; these averages were considered to represent the handgrip maximal voluntary force. To estimate the relative responsiveness levels of the M1 to voluntary drive during sustained maximal voluntary contractions, the force produced by the superimposed twitch (i.e., the twitch force) after TMS was expressed as a fraction of the pre-stimulus force at each TMS (Fig. 3) [4].

### 2.7 TMS

To measure MEPs, we used a method described in a previous study for examining the effects of unconscious goal pursuit on the motor system state [13]. In the pre-intermittent MVC and sustained MVC tasks, single, monophasic TMS pulses were applied to the left M1 (controlling the right hand) using a stimulator (M2002; Magstim, Whitland, UK) with a double-figure-eight-shaped coil (4150-00 Double 70 mm Alpha Coil; Magstim) and a maximum magnetic field strength of 1.55 T. All participants sat in an upright position with their elbows bent in front of them and their hands resting on their thighs. To identify the area that was evoking the largest response from the FCU muscle (i.e., the hot spot), the participants’ M1 was mapped extensively based on 10–15 stimuli while the coil was perpendicular to the anatomically defined central sulcus. The TMS coil was then manually positioned over the hot spot of the left M1, which was considered to have the lowest resting motor threshold (rMT). This area was defined as one in which MEPs with peak-to-peak amplitudes >50 μV were induced in the FCU muscle [38,44] in at least five of 10 trials when the participants were fully relaxed with their eyes closed [45]. The coil orientation was 45° lateral from the posterior–anterior electric field orientation. During the recording of the MEPs, the participants were instructed to remain in a resting state. A coil stand constructed of multiple products (Manfrotto Distribution KK, Tokyo, Japan) was used to stabilize the coil position throughout the experiment. A black marker pen was used to mark the optimal M1 position directly onto the scalp. To maintain consistent positioning throughout the experiment, the positioned coil was monitored continuously. Subsequently, the rMTs ranged from 40 % to 70 % of the maximum stimulator output.

Before the start of the experimental tasks, the stimulus intensity was changed in 10 % increments (from 45 % to 75 % of the maximum stimulator output) to determine the applied stimulus intensity in the pre-intermittent and sustained MVC tasks; this was sufficiently high to discriminate a single MEP waveform from bEMG activity [38]. The applied stimulus intensity was 73.3 % ± 1.1 % of the maximum stimulator output, which was equivalent to 127.0 % ± 3.7 % of the rMT; the stimulus intensity was not uniformly determined according to the stimulus intensity normalized to the rMT. The motor system state, which includes the level of M1 neuron recruitment, is unequal during MVC, resulting in a difference in the additional force evoked by cortical stimulation [4]. The stimulus intensity was constant for all participants under all conditions, and the obtained measurements were analyzed and compared within participants. Stimulation was delivered once manually over the target site during each brief MVC (duration: 2–3 s), with a 60-s inter-squeeze interval in the pre-intermittent MVC task (Fig. 2A and B). The stimulation was automatically delivered 50 times in the sustained MVC task (Fig. 3). Therefore, the MEPs in the sustained MVC task were recorded 50 times.

Surface electromyography (EMG) data were obtained from the right FCU muscles through bipolar silver surface electrodes (diameter: 10 mm; Nihon Kohden, Tokyo, Japan) using the tendon–belly method [38] and a constant inter-electrode distance of 20 mm. The skin over the identified muscles was wiped with alcohol prior to the placement of the electrodes. Signals (analysis time: 30 ms) were amplified using a bandpass filter (15 Hz–10 kHz) and digitized (MEG-6108; Nihon Kohden) at a sampling rate of 4 kHz.

### 2.8 Electrophysiology measurement and analysis

The peak-to-peak amplitude of each MEP, which reflects corticospinal excitability, was measured [46,47] (Fig. 2C) and the averaged MEP waveforms under the pre-intermittent MVC (average of three recordings) and sustained MVC tasks (average of 50 recordings) were calculated. To measure bEMG activity, a rectified EMG signal (approximately 100 ms before TMS) was integrated, with the force maintained at the maximum level (Fig. 2A and B). These analyses were performed using commercially available software (LabChart 7.3.8; ADInstruments, Tokyo, Japan).

The silent period was considered the time interval from the stimulus artifact to the return of continuous EMG [33,48] (Fig. 2C). It was difficult to determine the end of the silent period because voluntary EMG activity recovers gradually as opposed to abruptly, so it was determined as the moment that the corresponding rectified EMG activity reached a value within two SDs in the period approximately 100 ms before TMS [49,50] with careful visual inspection.

### 2.9 Statistical analysis

A repeated-measures one-way ANOVA of the experimental group (within-participants factor: [control or priming or priming + reward]) was conducted to analyze the maximal voluntary force, twitch force, silent period duration, MEP, bEMG, and pupil area under the sustained MVC task. Greenhouse–Geisser corrections were applied to adjust for non-sphericity, with a correction coefficient used to change the degrees of freedom. The Bonferroni correction for multiple comparisons was used in the post-hoc analysis. A *p*-value <0.05 was considered to indicate statistical significance in all tests. Partial η^2^ (η_p_^2^) is also shown as an effect size index when the results of the main effect and interaction of the one-way ANOVA are presented. Small, medium, and large effects were identified based on η_p_^2^ values of 0.1–0.24, 0.25–0.39, and ≥0.4, respectively [51]. All data are expressed as the mean ± standard error of the mean unless stated otherwise.

## 3 Results

### 3.1 Handgrip force

**Fig. 4:**
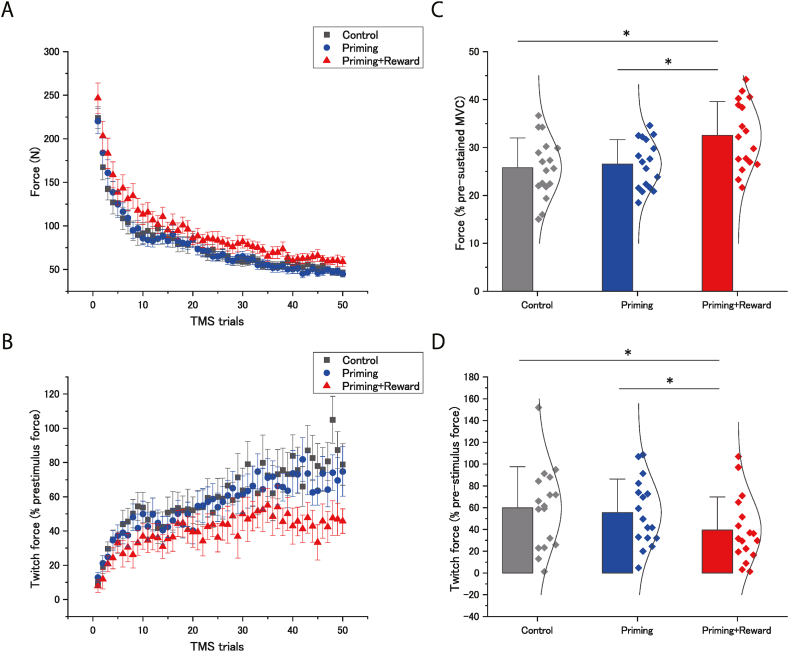
Effects of unconscious goal pursuit on the maximal voluntary force of handgrip. The maximal voluntary force for each condition (control, priming, and priming-plus-reward) during the maximal voluntary contraction (MVC) of handgrip in the sustained MVC task. Barely visible goal-priming with motivational reward significantly increased the maximal voluntary force of handgrip and significantly decreased the maximal voluntary force produced by the superimposed twitch (twitch force) following transcranial magnetic stimulation (TMS) during the sustained MVC. (A) The maximal voluntary force for each condition (control, priming, and priming-plus-reward) during the maximal voluntary contraction (MVC) of handgrip in the sustained MVC task. Data are expressed as the mean ± standard error of the mean. (B) The twitch force for each condition (control, priming, and priming-plus-reward) during the maximal voluntary contraction (MVC) of handgrip in the sustained MVC task. Data were expressed as a fraction of the pre-stimulus force at each TMS (mean ± standard error of the mean). (C) (D) Data were expressed as a percentage of the maximal voluntary force produced in pre-sustained MVC task. Barely visible goal-priming with motivational reward significantly increased the maximal voluntary force during the sustained MVC. Bar height shows the average across participants, the error bar represents one standard deviation, and the curve shows the normal distribution curve of individual plots. ∗Statistically significant difference between conditions.

Significant changes in maximal voluntary force during sustained MVC were observed in the priming-plus-reward condition (Table 1; Fig. 4A–C). One-way ANOVA revealed a significant difference between conditions (*F* [2,31] = 24.7; *p* < 0.001; effect size: η^2^_p_ = 0.61). Post-hoc analyses revealed that the force in the priming-plus-reward condition was significantly greater than those in the control (*p* < 0.001; effect size: *d* = 1.01) and priming (*p* < 0.001; effect size: *d* = 0.97) conditions (Fig. 4C). Regarding the twitch force (Fig. 4B–D), one-way ANOVA revealed a significant difference between conditions (*F* [2,31] = 6.91; *p* = 0.003; effect size: η^2^_p_ = 0.30). Post-hoc analyses revealed a significantly smaller twitch force in the priming-plus-reward condition than in the other conditions (comparison with control condition, [*p* = 0.004; effect size: *d* = 0.62]; comparison with the priming condition, [*p* = 0.027; effect size: *d* = 0.54]).

**Table 1:**
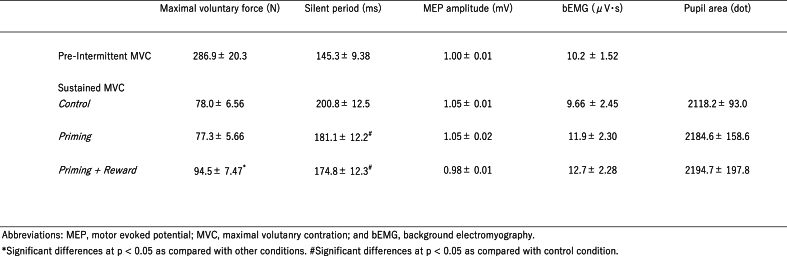
Maximal voluntary force, Silent period, MEP amplitude, bEMG and pupil area for the two tasks.

### 3.2 TMS

**Fig. 5:**
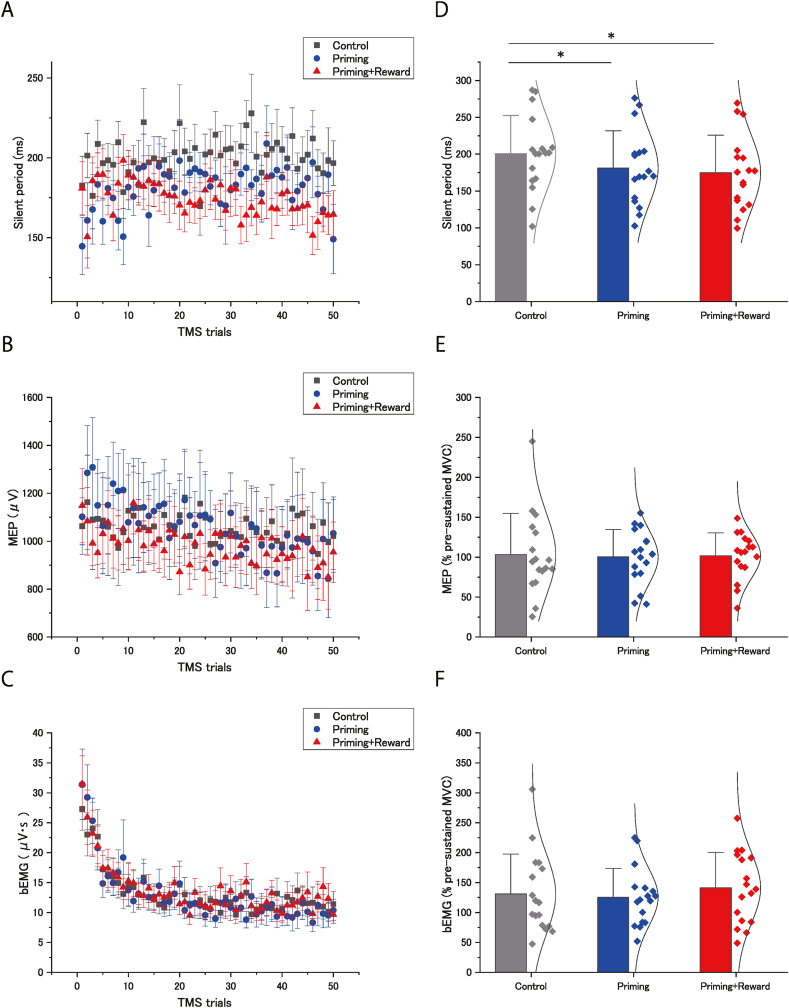
Effects of unconscious goal pursuit on the silent period, motor evoked potential (MEP) amplitude, and background (b) EMG. (A) The silent period, (B) amplitudes of MEPs of the flexor carpi ulnaris (FCU), and (C) bEMG activity of the FCU for each condition (control, priming, and priming-plus-reward) during handgrip maximal voluntary contraction (MVC) in the sustained MVC task. Data are expressed as the mean ± standard error. (D) The silent periods for the three conditions during handgrip MVC in the sustained MVC task. (E) Averaged amplitudes of 50 MEPs of the FCU for the three conditions during handgrip MVC in the sustained MVC task are shown as a percentage of those of three MEP amplitudes (an average of three recordings) during handgrip MVC in the pre-intermittent MVC task. (F) bEMG activity of the FCU for the three conditions during sustained MVC are shown as a percentage of those of three bEMG activities (an average of three recordings) during handgrip MVC in the pre-intermittent MVC task. Barely visible goal-priming with motivational reward significantly shortened the silent period during sustained MVC. Bar height shows the average across participants, error bars represent one standard deviation, and the curve shows the normal distribution curve of individual plots. ∗Significant difference between conditions.

Only the silent period (*F* [2,31] = 9.03; *p* < 0.001; effect size: η^2^_p_ = 0.36) (Table 1; Fig. 5A–D) differed according to condition, whereas the MEP amplitude (*F*[1.19,19.15] = 0.19; *p* = 0.71; effect size: η^2^_p_ = 0.012) (Table 1; Fig. 5B–E) and bEMG (*F* [2,31] = 0.66; *p* = 0.52; effect size: η^2^_p_ = 0.04) (Table 1; Fig. 5C–F) did not. The silent period in the priming-plus-reward (*p* < 0.001; effect size: *d* = 0.51) and priming (*p* = 0.012; effect size: *d* = 0.39) conditions were significantly shorter than that in the control condition, but no significant difference was found between the priming-plus-reward and priming conditions (*p* = 1.00; effect size: *d* = 0.12).

### 3.3 Pupil area

**Fig. 6:**
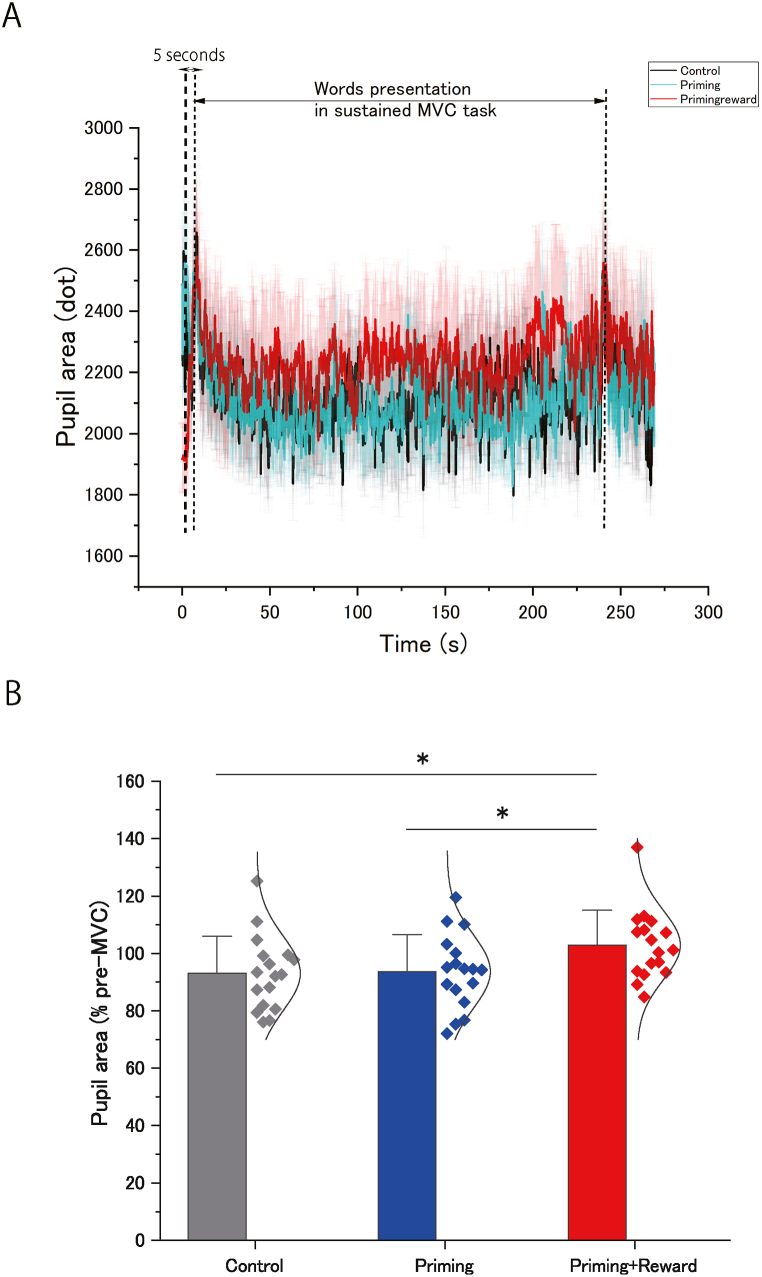
Effects of unconscious goal pursuit on pupil area. (A) Time course of pupil area (dots) expressed as the mean for each condition (control, priming, and priming-plus-reward) starting at the onset of priming word presentation for the three conditions during the maximal voluntary contraction (MVC) of handgrip in the sustained MVC task and lasting until approximately 32 s after the sustained MVC (mean ± standard error of the mean). The data were low-pass filtered with a cutoff frequency of 1 Hz using a fourth-order Butterworth filter. The bidirectional arrow (↔) with “5 s” indicates the period of 5 s immediately before the word presentation in the sustained MVC task. (B) Averaged pupil area (dots) for 500 ms before each transcranial magnetic stimulation while squeezing a handgrip device for the three conditions during the MVC of handgrip in the sustained MVC task was expressed as a percentage of mean pupil area during the 5 s period immediately before the word presentation for the three conditions. Barely visible goal-priming with motivational reward significantly increased pupil area during sustained MVC. Bar height shows the average across participants, the error bars represent one standard deviation, and the curve shows the normal distribution curve of individual plots. ∗Statistically significant difference between conditions.

Significant changes in the average pupil area were observed in the priming-plus-reward condition (Table 1; Fig. 6A), which was, in the following statistical analysis, normalized with that for 5000 ms before the priming word presentation in the sustained MVC task. One-way ANOVA revealed a significant difference between conditions (*F* [2,31] = 4.67; *p* = 0.017; effect size: η^2^_p_ = 0.23). Post-hoc analyses revealed a significantly greater pupil area (compared with the control condition, [*p* = 0.031; effect size: *d* = 0.77]; compared with the priming condition, [*p* = 0.046; effect size: *d* = 0.73]) in the priming-plus-reward condition compared with those in the other conditions (Fig. 6B).

## 4 Discussion

The present study aimed to investigate the effects of unconscious goal pursuit on the motor system state, the pupil-linked neuromodulatory system state, and maximal voluntary force by presenting motivational rewards during the manipulation of barely visible goal-priming cues while the participants performed sustained MVC. The results indicated that unconscious goal pursuit can elevate the force level of MVC via reduced motor cortical suppression, possibly via the potentiation of reward-connected dopaminergic system activity during sustained MVC. These results indicate that the muscular force-potentiating effect of unconscious goal pursuit in sustained handgrip force production with maximal effort is related to the potentiation of motor system activity caused by the additional drive of unconscious goal pursuit functioning on the M1.

The muscular force-potentiating impact of unconscious goal pursuit was followed by a decreased silent period. Silent periods longer than 100 ms in the hand muscles of able-bodied persons [52] reflect cortical suppression [48,[53], [54], [55]] and are utilized as an index for evaluating motor excitability [6]. Thus, unconscious goal pursuit appeared to enhance motor cortical activity. This possibility is favored by the decrease in superimposed twitch force in response to TMS applied over the M1 because a decreased twitch force indicates an increase in neurons and/or neuronal activity in the M1, which affects handgrip MVC [43]. Thus, a decreased twitch force implies an increased response of the M1 to maximal voluntary effort because lethargizing muscular contraction elevates the silent period without any changes in motoneuronal activity [56]. Therefore, we consider that the reduction of the silent period reflects a reduction of motor cortical suppression (i.e., the enhancement of motor cortical activity).

In spite of the decreased silent period, no changes in MEP amplitudes were found among the three conditions during handgrip sustained MVC (Fig. 5B–E). The reason for the unchanged MEP amplitudes may be that most of the M1 neurons had already been mobilized [57], resulting in fewer neurons being available to respond to TMS. Thus, any differences in MEP amplitudes during sustained MVC among the three conditions might have been overshadowed because the level of M1 neuron recruitment was leveling off during sustained MVC. However, it should be noted that a precondition for the interpretation of alternations in supraspinal fatigue without any detectable changes in MEP amplitude is that there were similar peripheral properties of the neuromuscular system across all three conditions.

A previous study reported a significant correlation between MEP amplitude and duration in a curvilinear regression model [58]. Thus, the reduced silent period (silent period onset = TMS artifact) cannot be explained by MEP duration alone because MEP amplitudes were unchanged across all three conditions, as mentioned above. Furthermore, considering that the MEP duration was approximately 40 ms (Fig. 2C), it is unlikely that the reduction in the silent period by approximately 26 ms under the priming + reward condition (Table 1) was caused by changes in MEP duration.

Barely visible primes related to rewards and the reward-dependent learning processed by dopaminergic activity, such as that in the ventral pallidum [59,60], can positively invigorate individuals to increase the effort they put into action [27]. Moreover, the coactivation of the detection function for positive reward signals in subcortical regions, along with the preparatory function of action at the perceptual, sensory, and motor levels based on an ideomotor principle, may play a key role in the affective-motivational effect of unconscious goal pursuit [41]. Positive affect alone has been found to motivate behavior only when associated with the representation of a particular behavior [61].

Subcortical regions such as the BG and thalamus [62,63] and motor-related brain areas [64,65] relate to the unconscious motivational process in cognitive control and decision-making, which is also linked to higher-level areas such as the anterior cingulate cortex [[66], [67], [68]]. In addition to the prefrontal cortex, the dorsal premotor cortex (PMd) is closely implicated in motor preparation and initiation [69], which play a strategic role in (movement) inhibition [70]. The PMd modulates stopping performance, in which human sensorimotor interaction necessitates not only mutual behavioral adaptation, but also shared cognitive task representations [71]. A previous study proposed a key role of premotor areas in the reward circuitry supporting action in which PMd neuronal activity represents a neuronal correlate of the impact of reward information on motor decisions [72]. Another study suggested that the PMd influences the M1 during response preparation in a variety of behavioral task contexts [73]. Thus, PMd neuronal activity might be implicated in the reducing impact of unconscious goal pursuit on motor cortical inhibition.

According to this neurophysiological alteration, in the present study, the prime-plus-reward stimuli enhanced the activity of motor cortical neurons. This result is also consistent with a previous report regarding the effects of unconscious goal pursuit on non-fatiguing brief handgrip MVC with enhanced corticospinal excitability [13]. 10.13039/100014337Furthermore, such a contribution of activity of the dopaminergic system to the activity of M1 neurons as assessed by the silent period is supported by previous research regarding the effects of motor neurological disorders on the silent period, in which the administration of the dopamine precursor levodopa l-3,4-dihydroxyphenylalanine was found to modulate the reduced silent period in Parkinson's disease by affecting neural activity in the 10.13039/501100024582BG, with specific effects on the inhibition of GABAergic receptors [74,75]. Additionally, intrathecal injection of GABAergic agonists has been shown to elongate the shorter silent period in patients with focal hand dystonia [55]. Therefore, the decrease of motor cortical suppression during sustained MVC may be caused by the additional drive of unconscious goal pursuit functioning on the M1 in the cortico-basal ganglia-thalamo-cortical loop.

The present findings revealed that the pupil area increased while viewing barely visible priming words with a reward. Although the direct influence of activity in noradrenergic LC neurons on pupil size is not well understood, noradrenergic neurons have been shown to be active during pupillary dilation [76]. Therefore, unconscious goal pursuit may potentiate pupil-linked neuromodulatory system activity through previously proposed neurophysiological processes [26,27], by which potentiated pupil-linked noradrenergic system activity may be caused by enhancement of dopaminergic system activity on the basis of the functionally and anatomically close associations between the noradrenergic and dopaminergic systems. It should be noted that the present results did not establish a causal link between the pupil-linked neuromodulatory system (pupillary diameter) and dopaminergic system activity.

No increase in the pupil area was observed under the priming condition in the present study [26]. However, the silent period under the priming condition was significantly reduced in the same manner as under the priming-plus-reward condition, relative to that under the control condition. This may have occurred because the priming condition did not shape the association between physical exertion-related words and motivational rewards (unconscious goal pursuit); barely visible exertion primes and positive words were both displayed but never paired, whereas the priming words were paired with neutral words. In other words, only one (preparation of action) of the basic processes necessary for goal pursuit mentioned above would be expected to operate in the priming condition without coactivation with the other process (detection of a positive reward signal). The process of unconscious preparation action on the basis of an ideomotor principle [77] is considered to be associated with motor-related brain areas [78,79]. Therefore, the reduction of the silent period in the priming condition would be expected to be induced by the additional drive of barely visible exertion primes operating on the M1 through the motor-related cortical areas. This result also supports our hypothesis that an additional drive in the M1 through motor-related cortical areas causes a motor cortical fatigue-diminishing effect during sustained MVC, as proposed by Takarada and Nozaki [5].

There are primary limitations to the generalization of the MVC results in this study. Although we did not evaluate the participants’ psychological or motivational stage in this study, we believe that the psychological or motivational stage was similar across the three conditions. The level of subjective effort was nearly identical in the three conditions, as evaluated by a category-ratio scale used in a previous study [13]. Thus, the observed differences in the force level of MVC were mainly caused by the connection between a motor-goal representation and positive affect in the subconscious process. However, care should be taken when interpreting the muscular force-potentiating effect because we could not completely eliminate the potential effects of condition order (control; priming; priming + reward) in the sustained MVC task from the muscular force-potentiating effect. Thus, whether the participants fully recovered for the second and third conditions by one instance of three brief MVCs in the pre-intermittent MVC task as a baseline for the sustained MVC task remains unclear.

In practice, many people experience emotional ups and downs when working out at the gym or undergoing rehabilitation at a rehabilitation institute. To achieve the same desired outcomes under such variable emotional conditions, the present findings suggest that gym and sports trainers and/or physical therapists, in addition to coaches, may be able to provide people with more positive advice and surroundings to elicit positive affective responses at their facilities by making use of the muscular force-potentiating effects of unconscious goal pursuit on a sustained fatiguing voluntary contraction including maximal force production via the presentation of positive words combined with the representation of a particular behavior with or without awareness, because they should sometimes make one last effort as they accept their limitations, regardless of exercise load.

In conclusion, the present findings revealed that barely visible goal-priming with motivational rewards resulted in a decreased silent period during sustained maximal force production and elevated handgrip maximal voluntary force. The elevated handgrip maximal force may have been caused by decreased motor cortical suppression during maximal effort induced by the additional drive of unconscious goal pursuit functioning on the motor system, possibly through the enhancement of reward-associated dopaminergic system activity. These findings indicate that an additional drive operating on the M1 has a motor cortical fatigue-diminishing effect during sustained maximal force production. In other words, the activity of the M1 during maximal force production in fatiguing maximal effort is affected by not only events in the M1 itself, but also input in one of the segmented BG-thalamocortical circuits to the M1.

## CRediT authorship contribution statement

**Yudai Takarada:** Writing – review & editing, Writing – original draft, Visualization, Validation, Supervision, Software, Resources, Project administration, Methodology, Investigation, Funding acquisition, Formal analysis, Data curation, Conceptualization. **Daichi Nozaki:** Writing – review & editing, Software.

## Ethics statement

The Human Research Ethics Committee of the Faculty of Sport Sciences of Waseda University approved the experimental procedures (approval No.: 2020-411), all of which complied with relevant laws and institutional guidelines.

## Data availability statement

Data will be made available on reasonable request.

## Declaration of competing interest

The authors declare that they have no known competing financial interests or personal relationships that could have appeared to influence the work reported in this paper.
